# The complete mitochondrial genome of *Bionychiurus tamilensis* (Collembola: Onychiuridae)

**DOI:** 10.1080/23802359.2026.2659972

**Published:** 2026-04-25

**Authors:** Han Soo Kim, Yujin Choi, Jeongwon Choi, Hyun-Gi Min, Soyeon Kwon, Byung Rae Jin, Yun Hui Kim, Ji Hyun Woo, Lee-Hyeon Jeon, Taekjun Lee, Yun-Sik Lee

**Affiliations:** a Department of Biology Education, Pusan National University, Busan, Republic of Korea; b Department of Animal Resources Science, Sahmyook University, Seoul, Republic of Korea; c Marine Animal Biodiversity Center, Sahmyook University, Seoul, Republic of Korea; d Institute for Future Earth, Pusan National University, Busan, Republic of Korea; e College of Natural Resources and Life Science, Dong-A University, Busan, Republic of Korea

**Keywords:** Collembola, springtail, gene order, phylogeny, mitogenome, *cox1* gene, *rrnS* gene

## Abstract

The complete mitochondrial genome of *Bionychiurus tamilensis* Thunnisa & Sumithra, 2021 was sequenced, assembled, and annotated. The mitochondrial genome of *B. tamilensis* is 14,937 bp in length and contains 13 protein-coding genes (PCGs), 22 transfer RNA (tRNA) genes, and two ribosomal RNA (rRNA) genes. The overall nucleotide composition was 35.3% A, 15.8% C, 9.8% G, and 39.6% T, indicating an obvious A + T bias (74.9%). *B. tamilensis* was closely clustered with the following species of Onychiuridae: *Allonychiurus kimi*, *Tetrodontophora bielanensis*, *Onychiurus orientalis*, and *Orthonychiurus folsomi*.

## Introduction

Collembola are distributed worldwide, comprising approximately 9500 described species and representing a diverse hexapod lineage that accounts for an estimated 32% of all terrestrial arthropods on Earth (Potapov et al. 2024). In soil ecosystems, these organisms function as decomposers, playing essential roles in organic matter breakdown and carbon and nitrogen cycling processes (Filser 2002). The family Onychiuridae represents a lineage specialized for subterranean environments, characterized by the absence of ocelli and furcula, shortened appendages, and well-developed pseudocelli and sensory setae (Greenslade and Ireson 2022). Recently, species in this family have been increasingly utilized as bioindicators for evaluating soil ecotoxicity due to their limited mobility, which facilitates handling and prevents avoidance behavior in response to contaminants (Min et al. 2025). However, despite these distinctive morphological characteristics and their importance in ecological studies, phylogenetic relationships among lineages within Onychiuridae remain inadequately resolved, primarily due to the scarcity of molecular sequence data suitable for phylogenetic analyses (Babenko et al. 2017). In the present study, we report the complete mitochondrial genome sequence of *Bionychiurus tamilensis* Thunnisa & Sumithra, 2021, representing the first mitogenomic data for this species, to establish its phylogenetic position within Onychiuridae and contribute molecular resources for future evolutionary and ecological studies of this family.

## Materials and methods

### Test specimens

**Figure 1.**
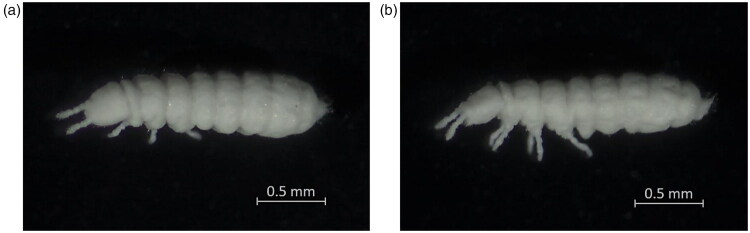
Reference images of *Bionychiurus tamilensis* collected from Mt. Seunghaksan in Busan, Republic of Korea (35°06′N, 128°59′E). (A) Dorsal view (adult). (B) Lateral view. Images were captured using a ZEISS Stemi 508 stereo microscope (Oberkochen, Germany) by Han Soo Kim in April 2025. Scale bars: 0.5 mm.

Specimens of *B. tamilensis* (Figure 1) were initially collected from Seunghaksan Mountain in Busan, Republic of Korea (35°06′N, 128°59′E) in April 2025. Subsequently, *B. tamilensis* has been maintained in laboratory culture for approximately six months. The DNA and voucher specimens used in the present study were deposited at Pusan National University (https://sites.google.com/view/ee-lab/home, su9778@pusan.ac.kr, specimen accession number: PNUCOL004).

### DNA extraction and mitogenome sequencing, assembly, and annotation

Total genomic DNA was extracted from whole bodies pooled from five adult specimens using a DNeasy^®^ Blood & Tissue Kit (Qiagen, Hilden, Germany), following the manufacturer’s protocol. Genomic DNA was further amplified using the random primers (Thermo Fisher Scientific, Cleveland, OH) and REPLI-g Mitochondrial DNA Kit (Qiagen, Hilden, Germany). Next-generation sequencing (NGS) was performed by the genome analysis facility at the National Instrumentation Center for Environmental Management (NICEM), Seoul National University, Seoul, South Korea. A genomic DNA library was constructed using the KAPA Hyper Prep Kit (Kapa Biosystems, Woburn, MA) and sequenced with paired-end reads on the Illumina Nextseq1000 platform (Illumina Inc., San Diego, CA). The mitogenome was assembled using a two-step process in Geneious Prime v.2025.0.3 (Biomatters Ltd, Auckland, New Zealand). First, raw sequencing reads were processed for quality trimming and pre-assembled using the *de novo* assembly method to generate initial contigs. Subsequently, these contigs were refined and validated using the map-to-reference function. The final mitogenome sequence was determined based on the consensus derived from this reference-guided mapping, with sequencing depth and coverage detailed in Figure S1. The coverage depth was calculated using SAMtools (Danecek 2021) from BAM files exported from Geneious Prime, and visualized in RStudio (R Core Team 2025). Genes were annotated using GeSeq (Tillich et al. 2017) and MITOS2 in GALAXY web server (The Galaxy Community 2024). Incomplete stop codons (T– or TA−) were assumed to be completed to functional TAA stop codons by post-transcriptional polyadenylation, following the mitochondrial transfer RNA (tRNA) punctuation model (Faure and Barthélémy 2018). The circular mitochondrial genome map of *B. tamilensis* was created using Proksee web server (Grant et al. 2023). AT-skew and GC-skew were calculated according to the formulas AT-skew = [A – T]/[A + T] and GC-skew = [G − C]/[G + C], following Perna and Kocher (Sun 2020; Perna and Kocher 1995).

### Phylogenetic analysis

For the phylogenetic analysis, nucleotide sequences of the 13 mitochondrial protein-coding genes (PCGs) from 23 Hexapoda species were analyzed, including nine Poduromorpha, two Symphypleona, nine Entomobryomorpha, and two Diplura sequences obtained from NCBI, along with the *B. tamilensis* mitochondrial genome generated in this study. Nucleotide sequences of each PCG were aligned using MAFFT v7.450 (Katoh and Standley 2013), and the optimal substitution model for each aligned PCG was determined using jModelTest 2.1.10 (Guindon and Gascuel 2003; Darriba et al. 2012) (Table S1). Because some of the best-fit models identified by jModelTest are not implemented in MrBayes, we applied GTR or HKY models that also appeared among the top-ranked candidates in the BIC model-selection output for the BI analysis. Phylogenetic analysis was conducted using Bayesian inference (BI). The BI tree was analyzed using MrBayes 3.2.7 (Ronquist and Huelsenbeck 2003) with third codon positions excluded from the dataset, employing two independent MCMC runs of 1,000,000 generations each, sampling every 1000 generations, and discarding the first 250 samples as burn-in. Phylogenetic trees were visualized using FigTree v1.4.4 (Rambaut 2018).

## Results

### Complete mitochondrial genome structure of *B. tamilensis*

**Figure 2.**
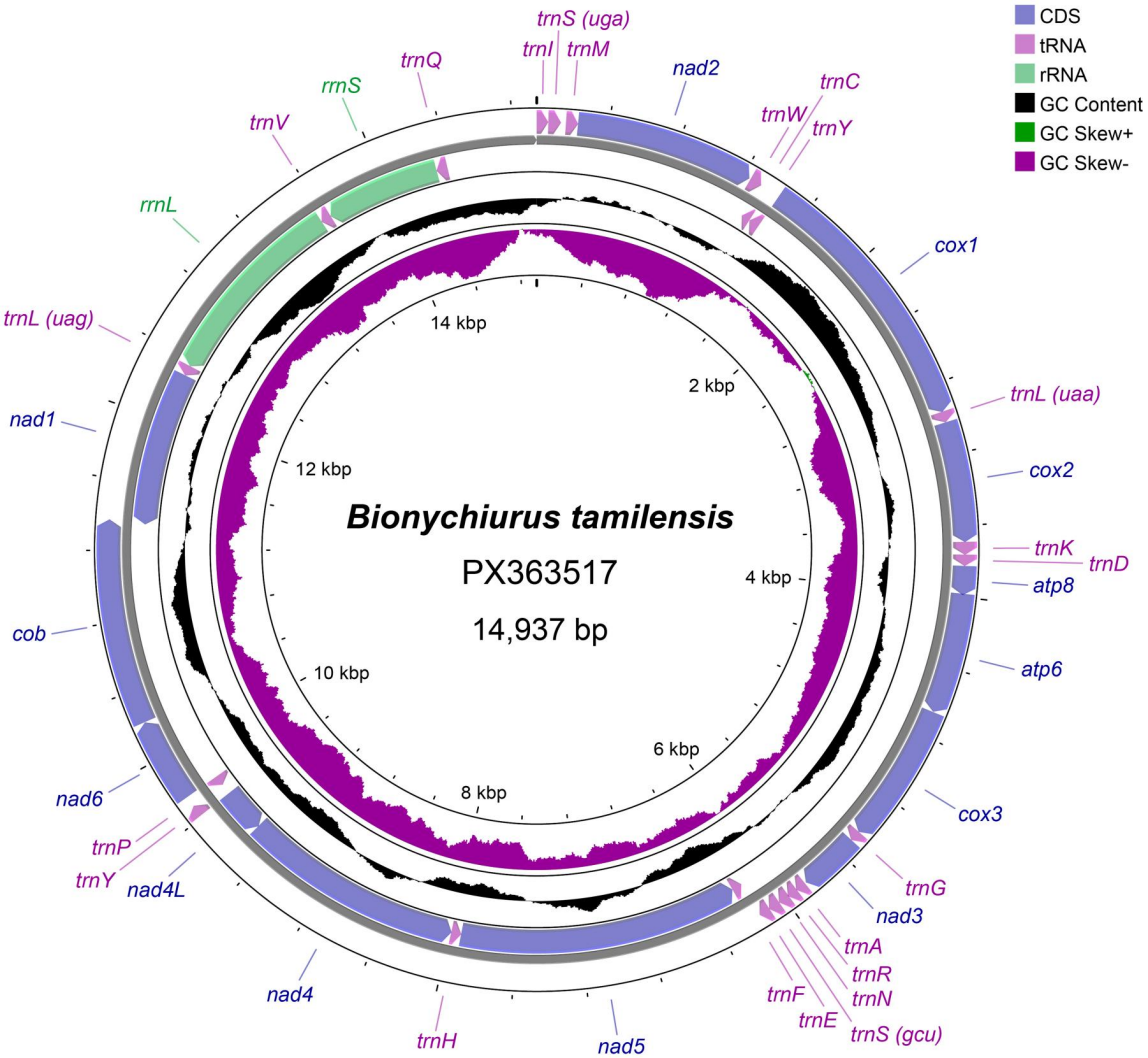
Structure of the mitochondrial genome of *B. tamilensis*. Genes encoded on the forward strand (H-strand) are shown on the outer circle, and those on the reverse strand (L-strand) are shown on the inner circle. The complete mitochondrial genome is 14,937 bp in length. Protein-coding genes (CDS), transfer RNA genes (tRNAs), and ribosomal RNA genes (rRNAs) are indicated in blue, pink, and light green, respectively. The inner black circle represents GC content, and the colored plot indicates GC skew, with positive values shown in green and negative values shown in purple. Local compositional indices (GC content and GC skew) were calculated using a sliding window approach with a window size of 500 bp and a step size of 10 bp.

The complete mitochondrial genome of *B. tamilensis* is 14,937bp in length (GenBank accession number: PX363517), comprising 13 PCGs, 22 tRNA genes, two ribosomal RNA (rRNA) genes (Figure 2). The overall nucleotide composition of the mitochondrial genome, calculated based on the heavy strand, is 35.3% of adenine (A), 39.6% of thymine (T), 9.3% of guanine (G), and 15.8% of cytosine (C), resulting in an AT-skew of −0.057 and a GC-skew of −0.259.

Among the 13 PCGs, six different start codons were observed: ATG (four genes), ATA (three genes), ATT (three genes) and TTG, AAA, ATC (one gene each) (Table S1). Nine PCGs terminate with the complete stop codon TAA, while four PCGs end with incomplete stop codons (T– or TA−). The 22 tRNA genes range from 53 bp to 72 bp in length. The *rrnL* and *rrnS* genes are 1197 bp and 701 bp in length, respectively. Intergenic spacers range from 2 bp to 28 bp, with the longest spacer (28 bp) located between *trnS1* and *trnM*. Thirteen gene pairs exhibit overlapping regions ranging from 1 bp to 15 bp, with the longest overlap (15 bp) occurring between *cob* and *nad1*.

### Phylogenetic analysis

**Figure 3.**
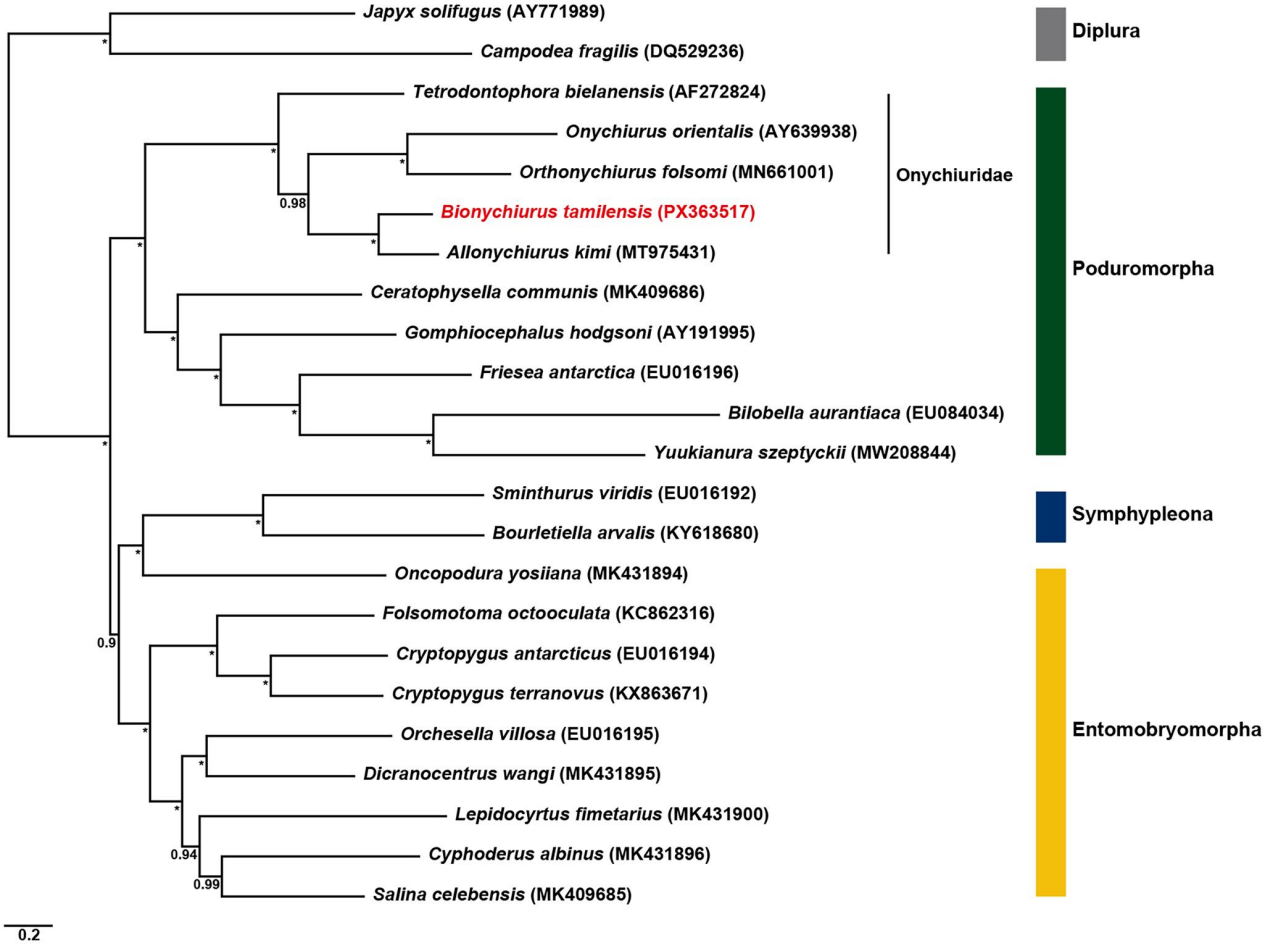
Phylogenetic tree using Bayesian inference (BI) method with the third codon positions removed based on the nucleotide sequences of 13 protein coding genes from 23 Hexapoda, including *B. tamilensis*, with two Diplura as the Entognatha outgroups. Support values are provided at internal nodes; posterior probabilities are indicated by asterisks (*) when they reach the maximum values 1.0, respectively. The sequences analyzed were as follows: AF272824 (Nardi et al. 2001); AY191995 (Nardi et al. 2003); AY639938 (Cook et al. 2005); AY771989 (Carapelli et al. 2005); DQ529236 (Podsiadlowski et al. 2006); EU016192, EU016194, EU016195, EU016196, EU084034 (Carapelli et al. 2007); KC862316 (Carapelli et al. 2014); KY618680, KX863671 (direct submission); MK409686 (Dong et al. 2020); MK409685, MK431894, MK431895, MK431896, MK431900 (Sun et al. 2020); MN661001 (Yao et al. 2020); MW208844 (Wee et al. 2021).

Bayesian inference phylogenetic tree was reconstructed based on concatenated sequences of 13 PCGs with third codon positions removed from 23 Hexapoda species, including *B. tamilensis*, with two Diplura (*Japyx solifugus* and *Campodea fragilis*) as Entognatha outgroups (Figure 3). The tree topology received strong support across most nodes, with posterior probabilities ranging from 0.90 to 1.00. Notably, the phylogenetic analysis showed distinct clustering of species according to their respective orders, placing *B. tamilensis* within the Poduromorpha lineage. *B. tamilensis* formed a strongly supported sister relationship (posterior probability = 1.00) with *Allonychiurus kimi* (MT975431). This clade further clustered with a subclade comprising *Orthonychiurus folsomi* (MN661001) and *Onychiurus orientalis* (AY639938), and with *Tetrodontophora bielanensis* (AF272824), forming a monophyletic Onychiuridae group with strong support (posterior probability = 1.00). These results support the phylogenetic placement of *B. tamilensis* within Onychiuridae and clarify its evolutionary relationship with related genera.

## Discussion and conclusions

This study presents the first complete mitochondrial genome sequence of *B. tamilensis*, which was recovered as a closely related species to *A. kimi* (MT975431; Lee et al. 2021). The mitogenome exhibits a typical gene arrangement and high AT bias (74.9%) consistent with other Collembola species (Yao et al. 2020; Lee et al. 2021). The presence of multiple start codon types and incomplete stop codons in several PCGs reflects the genomic characteristics commonly observed across hexapod mitogenomes, suggesting conservation of mitochondrial genome organization within Onychiuridae (Nardi et al. 2001; Carapelli et al. 2014).

Phylogenetic reconstruction using BI revealed that *B. tamilensis* forms a strongly supported sister relationship with *A. kimi* (posterior probability = 1.00). This clade further clusters with *Orthonychiurus folsomi* and *Onychiurus orientalis* and forms a monophyletic Onychiuridae group together with *T. bielanensis*, receiving strong support (posterior probability = 0.98–1.00). These findings are consistent with morphological classifications and support the phylogenetic placement of *B. tamilensis* within Onychiuridae. Onychiuridae species have long been utilized in soil ecotoxicity assessments due to their ecological characteristics. Recently, for instance, mitogenomic data from *A. kimi* enabled the development of species-specific eDNA primers, advancing the precision of species detection in environmental monitoring (Lee et al. 2022). The mitogenome of *B. tamilensis* reported here extends beyond phylogenetic reconstruction, providing molecular resources that will facilitate species identification, population genetics studies, and conservation biology applications. These findings establish a critical foundation for future ecological and evolutionary research on Onychiuridae and soil-dwelling Collembola.

## Supplementary Material

ARRIVE guidelines Author Checklist.pdf

Supplementary File.docx

Supplementary File_highlight.docx

## Data Availability

BioSample, BioProject, and SRA accession numbers are SAMN50461601, PRJNA1302118, and SRR34876616, respectively. The genome sequence data supporting the findings of this study are openly available in GenBank of the National Center for Biotechnology Information (NCBI), with an accession number PX363517.
