# Supplementary material for: The complete mitochondrial genome of Bionychiurus tamilensis (Collembola: Onychiuridae)
Source: Mitochondrial DNA B Resour. 2026 Apr 25;11(5):681–5. doi: 10.1080/23802359.2026.2659972 (PMC13112862; doi:10.1080/23802359.2026.2659972)
Supplement: Supplementary File.docx [file TMDN_A_2659972_SM3740.docx]

**Supplementary Material for:**

**The complete mitochondrial genome of *Bionychiurus tamilensis* (Collembola: Onychiuridae)**

Han Soo Kim^a,1^, Yujin Choi^b,d,1^, Jeongwon Choi^a^, Hyun-Gi Min^c^, Soyeon Kwon^d^, Byung Rae Jin^e^, Yun Hui Kim ^e^, Ji Hyun Woo^e^, Lee-Hyeon Jeon^a^, Taekjun Lee^b,d^, Yun-Sik Lee^a*^

^a^ Department of Biology Education, Pusan National University, Busan 46241, Republic of Korea

^b^ Department of Animal Resources Science, Sahmyook University, Seoul 01795, Republic of Korea

^c^ Institute for Future Earth, Pusan National University, Busan 46241, Republic of Korea

^d^ Marine Animal Biodiversity Center, Sahmyook University, Seoul 01795, Republic of Korea

^e^ College of Natural Resources and Life Science, Dong-A University, Busan 49315, Republic of Korea

*** Corresponding author:**

Yun-Sik Lee

Tel. 82-0515102791

e-mail: [yunsiklee@pusan.ac.kr](mailto:yunsiklee@pusan.ac.kr)

^1^These authors contributed equally to this work


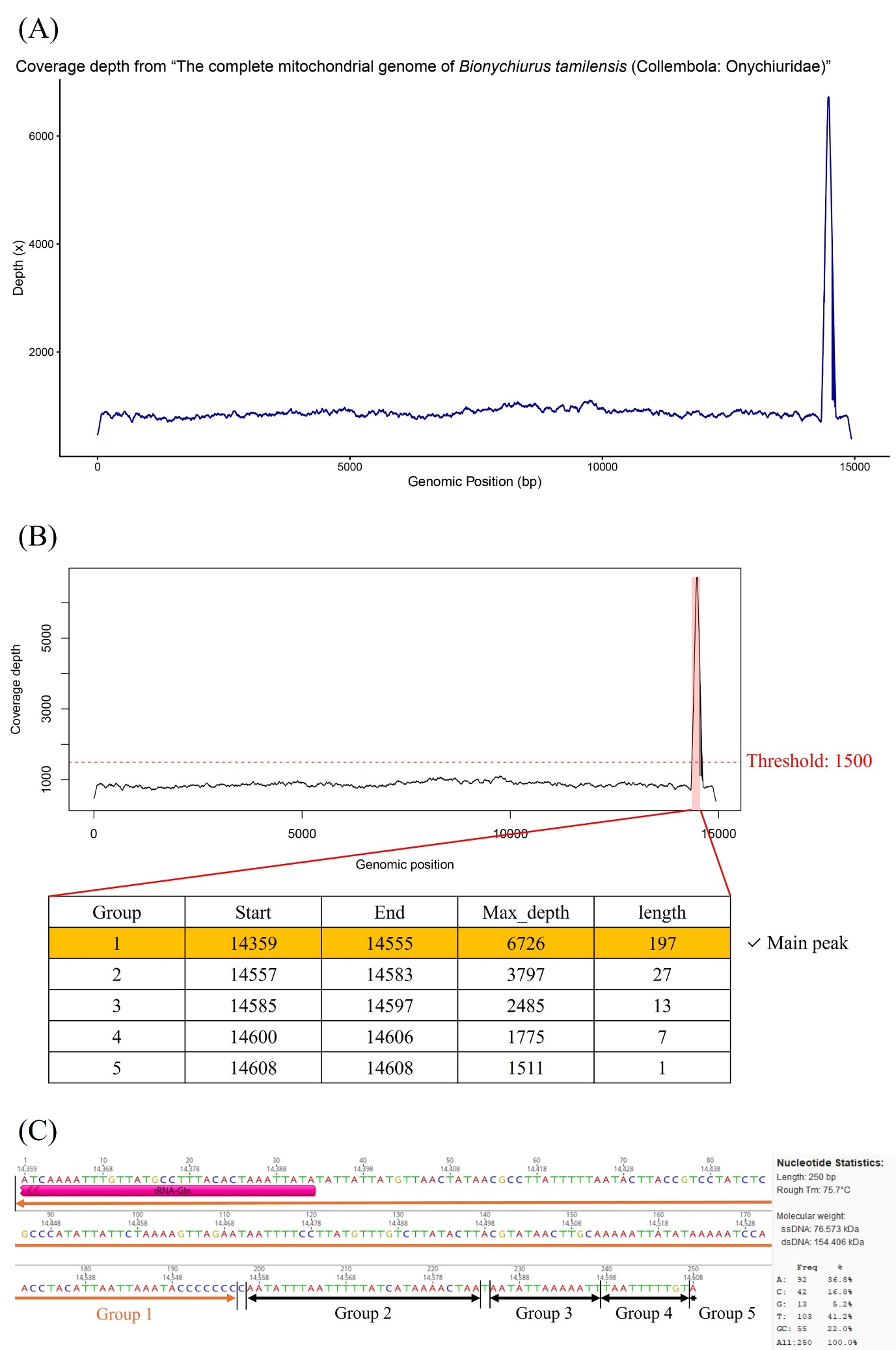


**Figure S1.** Coverage profile and characterization of high-depth regions in the mitochondrial genome of *Bionychiurus tamilensis*. (A) Genome-wide coverage depth across the mitochondrial genome. Coverage depth refers to the number of times a nucleotide sequence is read during assembly. The x-axis represents the genomic position, and the y-axis indicates the coverage depth. Coverage across most of the mitochondrial genome is highly uniform (approximately 700–1000×), indicating high sequencing quality and supporting the reliability of the assembly. A narrow coverage peak (~6726×) is observed in a localized region. (B) Identification and quantification of high-coverage regions based on threshold filtering. High-coverage groups were identified using a coverage threshold of 1500×, resulting in five continuous groups. Each group represents a continuous genomic region where coverage depth exceeds the defined threshold (1500×) without interruption. Among these, Group 1 (positions 14,359–14,555 bp; maximum depth 6726×; length 197 bp) corresponds to the main peak. (C) Nucleotide sequence of the region where coverage depth exceeds 1500× (positions 14,359–14,608 bp). The sequence is partitioned into five groups corresponding to the coverage-based grouping shown in panel (A). This region exhibits an overall AT-rich and low-complexity sequence composition, including short AT-rich motifs and homopolymer-like stretches.

**Table S1.** Annotated mitochondrial genes of *B. tamilensis* with gene boundaries, start/stop codons, anticodons (for tRNAs), and best-fit substitution models (BIC) for PCGs.

| **No** | **Name** | **Start Codons** | **Stop Codons** | **BIC** | **Anti codon** | **Min** | **Max** | **Strand** |  |
| --- | --- | --- | --- | --- | --- | --- | --- | --- | --- |
|  |  |  |  |  |  |  |  |  |  |
| 1 | *trnI* | - | - | - | GAU | 1 | 64 | + |  |
| 2 | *trnS2* | - | - | - | UGA | 66 | 135 | + |  |
| 3 | *trnM* | - | - | - | CAU | 164 | 230 | + |  |
| 4 | *nad2* | TTG | TAA | TVM+I+G |  | 231 | 1235 | + |  |
| 5 | *trnW* | - | - | - | UCA | 1244 | 1311 | + |  |
| 6 | *trnC* | - | - | - | GCA | 1309 | 1361 | - |  |
| 7 | *trnY* | - | - | - | GUA | 1364 | 1426 | - |  |
| 8 | *cox1* | AAA | T-- | TPM2uf+I+G |  | 1428 | 2955 | + |  |
| 9 | *trnL2* | - | - | - | UAA | 2955 | 3016 | + |  |
| 10 | *cox2* | ATT | T-- | TVM+I+G |  | 3017 | 3692 | + |  |
| 11 | *trnK* | - | - | - | CUU | 3692 | 3763 | + |  |
| 12 | *trnD* | - | - | - | GUC | 3763 | 3825 | + |  |
| 13 | *atp8* | ATC | TAA | GTR+I+G |  | 3826 | 3987 | + |  |
| 14 | *atp6* | ATG | TAA | TPM2uf+I+G |  | 3981 | 4658 | + |  |
| 15 | *cox3* | ATG | T-- | TVM+I+G |  | 4661 | 5447 | + |  |
| 16 | *trnG* | - | - | - | UCC | 5448 | 5508 | + |  |
| 17 | *nad3* | ATA | TAA | TVM+I+G |  | 5509 | 5853 | + |  |
| 18 | *trnA* | - | - | - | UGC | 5862 | 5921 | + |  |
| 19 | *trnR* | - | - | - | UCG | 5920 | 5979 | + |  |
| 20 | *trnN* | - | - | - |  | 5976 | 6039 | + |  |
| 21 | *trnS1* | - | - | - | GCU | 6040 | 6105 | + |  |
| 22 | *trnE* | - | - | - | UUC | 6105 | 6167 | + |  |
| 23 | *trnF* | - | - | - | GAA | 6167 | 6228 | - |  |
| 24 | *nad5* | ATA | TAA | TVM+I+G |  | 6230 | 7930 | - |  |
| 25 | *trnH* | - | - | - | GUG | 7931 | 7991 | - |  |
| 26 | *nad4* | ATG | TAA | TVM+I+G |  | 7991 | 9343 | - |  |
| 27 | *nad4L* | ATT | TA- | TVM+I+G |  | 9343 | 9611 | - |  |
| 28 | *trnY* | - | - | - | UGU | 9612 | 9677 | + |  |
| 29 | *trnP* | - | - | - | UGG | 9676 | 9737 | - |  |
| 30 | *nad6* | ATA | TAA | TPM2uf+G |  | 9739 | 10215 | + |  |
| 31 | *cob* | ATG | TAA | GTR+I+G |  | 10218 | 11372 | + |  |
| 32 | *nad1* | ATT | TAA | TVM+I+G |  | 11358 | 12299 | - |  |
| 33 | *trnL1* | - | - | - | UAG | 12300 | 12362 | + |  |
| 34 | *rrnL* | - | - | - |  | 12363 | 13559 | - |  |
| 35 | *trnV* | - | - | - | UAC | 13560 | 13623 | - |  |
| 36 | *rrnS* | - | - | - |  | 13624 | 14324 | - |  |
| 37 | *trnQ* | - | - | - | UUG | 14325 | 14392 | - |  |
